# Extracellular Vesicles of Mesenchymal Stem Cells Are More Effectively Accessed through Polyethylene Glycol-Based Precipitation than by Ultracentrifugation

**DOI:** 10.1155/2022/3577015

**Published:** 2022-09-06

**Authors:** Lei Jia, Bo Li, Cong Fang, Xiaoyan Liang, Yingjun Xie, Xiaofang Sun, Wen Wang, Lei Zheng, Ding Wang

**Affiliations:** ^1^The First School of Clinical Medicine, Southern Medical University, Guangzhou 510515, China; ^2^Department of Laboratory Medicine, Nanfang Hospital, Southern Medical University, Guangzhou 510515, China; ^3^Reproductive Medicine Center, The Sixth Affiliated Hospital, Sun Yat-sen University, Guangzhou 510275, China; ^4^Department of Obstetrics and Gynecology, Guangdong Provincial Key Laboratory of Major Obstetric Diseases, The Third Affiliated Hospital of Guangzhou Medical University, Guangzhou, 510150 Guangdong, China; ^5^Guangdong Provincial Key Laboratory of Major Obstetric Diseases, The Third Affiliated Hospital of Guangzhou Medical University, Guangzhou, 510150 Guangdong, China; ^6^School of Engineering and Materials Science, Queen Mary University of London, London E1 4NS, UK

## Abstract

Extracellular vesicles (EVs) have been identified as cell-cell communication agents, and EVs derived from mesenchymal stem cells (MSCs) exhibit therapeutic effects similar to those of the cells of origin. Precipitation methods have been used extensively for EV harvests, such as UC- (ultracentrifugation-) or PEG- (polyethylene glycol-) based methods, and the difference in EVs derived from MSCs by UC and PEG is not fully understood. We harvested EVs from amniotic fluid MSCs (AF-MSCs) by UC- or PEG-based precipitation methods and conducted a comparison study of those EVs derived by the two methods: output, RNA, and protein expression of EVs and EV biological reaction in a THP-1-cell model of LPS induction, which was considered an infection model. There was no difference in morphology, size, or specific marker-positive ratio of PEG-EVs and UC-EVs, but PEG obtained more EV particles, protein, and RNA than the UC method. In our THP-1 model of LPS induction, MSC-EVs did not lead to a change in protein expression but inhibited the LPS-induced increase in cytokine secretion. UC-EVs were more effective for TNF-*α* inhibition, and PEG-EVs were more effective for IL10 inhibition. Thus, our findings provide evidence that PEG-based precipitation is a more efficient mesenchymal stem cell-extracellular vesicle-derived method than UC.

## 1. Introduction

Mesenchymal stem cells (MSCs) are defined as multipotent cells with certain criteria [1]; however, recent in vivo and in vitro studies of MSC biological function have revealed that the most important biological function of exogenous MSC transplantation is immune activity regulation [2] via paracrine effects for antitissue damage [3, 4] or cancer therapy [5–7]. Although cytotherapy of MSCs was implemented by the administration of millions of cells, most of the grafted cells died once they crossed the physiological or pathological microenvironment of individuals who were receiving treatment [8, 9], and other therapeutic strategies were formulated by MSC-derived products, such as conditioned medium or extracellular vesicles (EVs) [4, 7]. MSC-derived EVs are apoptotic bodies, microvesicles, and exosomes [10] that can achieve MSC therapeutic effects while avoiding the side effects caused by sudden cell death and the potential tumorigenicity of surviving cells. The classic EV isolation method is ultracentrifugation (UC), and some commercial kits have been developed for EVs harvested from various kinds of liquid. The polyethylene glycol-based precipitation method [11] was characteristic of similar miRNA harvest, similar particle size, and higher protein harvest achieved compared with UC methods in plasma samples [12–14]. Nevertheless, there were significant differences in the shapes and protein types of EVs derived from saliva by these two methods (UC and PEG) [15]. The differences in MSC-EVs derived through UC and PEG are not well known, including their output and biological function.

MSCs can be derived from various tissues; while they meet the criteria of MSCs and have effective therapeutic effects in animal models, there are still some differences in morphology and biological function [16, 17]. The amniotic fluid cells (AFCs) of the second trimester could be cultured in dishes for several passages, which have been used for classical cytogenetic prenatal diagnosis [18]. In previous work, we identified AFCs as a kind of MSC (AF-MSC) that express MSC-specific biomarkers and can be induced to differentiate into mesodermal terminal cells and inhibit PHA- (phytohemagglutinin-) activated PBMC (peripheral blood mononuclear cell) division [19]. Furthermore, AFMSCs share similar biological effects with other sources of MSCs in vivo, as they can effectively inhibit acute immune activation caused by intestinal microorganism infection [20] or hyperoxia [21]. The inflammatory response can be mimicked by human myeloid leukemia mononuclear (THP-1) cells treated with lipopolysaccharide (LPS) in vitro [22]. Macrophage development induced by LPS is a complicated process, as M1-polarized macrophages are activated, while some alternatively activated macrophages are also induced, such as M2b [23]. EVs derived from MSCs perform anti-inflammatory functions by promoting M2 macrophage polarization [24].

MSCs have been identified as an effective treatment for injury-induced diseases [25] and cancer [26]. One of the accepted therapeutic theories for MSCs is that EVs derived from MSCs function in the tissue microenvironment, resulting in therapeutic effects. EVs generally describe particles of cellular origin. According to the molecular composition and source, they can be classified as ectosomes and exosomes [27] or classified as microvesicles, exosomes, and apoptotic bodies according to their biogenesis [28]. Exosomes specialize in particles that carry cellular cargo ranging from 40 to 120 nm and are functional components of EVs. EVs are considered transmitters of physiological and pathological processes [29], and EVs derived from MSCs display biofunctions similar to those of their source cells during the treatment of inflammatory diseases [4], cancer, and aging [7]. In a previous study, we found that AF-MSCs could inhibit the activation of immune cells in vitro [19] and in vivo [20, 21], and we expect to establish a simple, low-cost, and effective method to derive EVs from AFMSCs to prepare a feasible EV-based therapeutic schedule without MSC transplantation.

In the current study, we derived EVs from AF-MSCs through the UC and PEG methods and compared the physical and chemical characteristics of UC-EVs and PEG-EVs, as well as the effect on target cells. We designed an LPS-exposed THP-1-cell experiment to compare the biological functions of UC-EVs and PEG-EVs. UC-EVs and PEG-EVs shared similar morphology, size, and protein expression. PEG harvested more EV particles, protein, and RNA than the UC method, and there were differences in EV RNA expression and cytokine secretion inhibition levels in the cell model of THP-1 cells with LPS induction. Specifically, compared with PEG-EVs, the inhibitory effect of UC-EVs on TNF-*α* was stronger, while it was weaker for IL-10 inhibition after THP-1 was activated by LPS, suggesting that MSC-EVs obtained by different methods differ in the function of secreted factors produced by immune cells exposed to infection. Collectively, we demonstrated the difference in EV harvest methods, including UC- and PEG-based precipitation, and compared the effects of UC-EVs and PEG-EVs on RNA, protein, and cell biological effects in LPS induction of THP-1 cells.

## 2. Materials and Methods

### 2.1. Cell Culture

Human MSCs were isolated from amniotic fluid (AF-MSCs) of the second trimester, while pregnant women underwent cytogenetic analysis for clinical purposes. This study was approved by the Academic Committee of the Third Affiliated Hospital of Guangzhou Medical University, and the involved patients signed consent to participate in this study. MSCs were primarily cultured in commercial AFC medium and subcultured in Dulbecco's modified Eagle's medium (DMEM) with low glucose (Cat# C11885500BT, Gibco, Massachusetts, USA) supplemented with 20% fetal bovine serum (FBS) (Cat# 10099141, Gibco). Human myeloid leukemia mononuclear cells (THP-1), which were purchased from the Cell Bank of the Chinese Academy of Sciences, were maintained in RPMI 1640 (Cat# 11875, Gibco, Massachusetts, USA) supplemented with 10% FBS. All cells were passaged by trypsin (Cat# 25200072, Gibco, Massachusetts, USA) when the cells proliferated and reached over 90% confluence. The cell morphology was recorded by the Optec OPTPro V 4.7 software using a BK6000 microscope (Chongqing Optec Instrument Co., Ltd, Chongqing, China).

### 2.2. Extracellular Vesicle Harvest

EVs were harvested from passage 2 to passage 5 from AFMSCs. When the AFMSCs reached 70% confluence, the culture medium was changed to a non-FBS culture medium (DMEM with low glucose) for an extended 72-hour incubation. The medium for EV harvest of different cells and different passages was mixed into a total of 250 ml and equally divided into two parts for comparison of EVs isolated by UC or PEG. EV isolation through different methods was independently repeated three times for statistical analysis. Large vesicles were removed by centrifugation at 10,000 × g for 45 min at 4°C (Microfuge 20R, Beckman, Germany), and the supernatant was used for EV isolation. For UC, 125 ml of cell culture medium was centrifuged at 100,000 × g for 70 min at 4°C using an ultracentrifuge (CP100MX, Hitachi, Ltd., Tokyo, Japan) to preliminarily collect the EVs. The EV pellet was resuspended in 10 ml prechilled (at 4°C) PBS (phosphate-buffered saline) for the second ultracentrifugation of 100,000 × g for 70 min at 4°C. Finally, the pellet was resuspended in 500 *μ*l of precooled PBS for further study. For PEG, 125 ml cell culture medium was mixed with an equal volume of PEG 20000 (Cat# A601790-0250, Sangon Biotech, Shanghai, China) solution (16 g PEG in 100 ml 1 M NaCl) for 12 h at 4°C. Then, the mixed medium was centrifuged at 10,000 × g for 20 min at 4°C for EV pellet harvest (Microfuge 20R, Beckman). The pellet was resuspended in 500 *μ*l of precooled PBS as UC.

### 2.3. Quantitative Analysis of EV Proteins and RNA

Proteins in EVs were quantified by a BCA Protein Assay Kit (Cat# P0011, Beyotime Biotechnology, Shanghai, China) according to the manufacturer's instructions. EV RNA was quantified by a Qubit™ RNA HS Assay Kit (Cat# Q32852, Thermo Fisher Scientific, CA, USA) according to the manufacturer's instructions.

### 2.4. Nanoflow Cytometry Analysis

Nanoflow cytometry was used to determine the molecular marker expression and the concentration and size distribution of the EVs on a Flow NanoAnalyzer (N30E, NanoFCM INC., Fujian, China). For concentration and size distribution analysis, EV samples were diluted 1 : 100 in PBS and directly analyzed according to the Flow NanoAnalyzer's instructions. For molecular markers, EV samples were diluted 1 : 100 in PBS and labeled by fluorescent antibodies, including FITC Mouse Anti-Human CD9 (Cat# 555371, BD Biosciences, NJ, USA), FITC Mouse Anti-Human CD63 (Cat# 556019, BD Biosciences), FITC Mouse Anti-Human CD81 (Cat# 551108, BD Biosciences), and FITC Mouse IgG (Cat# 400108, BioLegend, CA, USA).

### 2.5. Transmission Electron Microscopy

The EVs were fixed with equal volume of paraformaldehyde (PFA) (4%). The fixed EVs (10 *μ*l) were dropped on a no copper grid and kept at room temperature for 1 min to ensure that the protein bonded to the bottom and that the liquid was removed with filter paper. Uranyl acetate was dripped into the sample area for 1 min and then removed with filter paper. The prepared sample was then dried at room temperature for further observation. Transmission electron microscopy was performed on a JEM-1400 PLUS (Japan Electron Optics Laboratory Co., Ltd., Tokyo, Japan). Images were captured by a VELETA G3 camera (EMSIS Münster, Germany), and the size of the EVs was determined by the RADIUS software (EMSIS).

### 2.6. LPS Stimulation and EV Function Analysis In Vitro

The infection was mimicked by exposure of THP-1 cells (10000/ml) to 1 *μ*g/ml LPS (lipopolysaccharide) for 4 h. Then, the source of infection was removed, and 20 *μ*g/ml EVs (1.9 × 108 particles/ml) derived from PEG or UC were added to the culture medium for 36 h of treatment. Finally, the culture medium and THP-1 cells were harvested for mRNA and protein analysis.

### 2.7. RNA Quantification by Droplet Digital Polymerase Chain Reaction (PCR)

Total RNA of cells and EVs was extracted using TRIzol (Cat# TR118, Molecular Research Center, Cincinnati, OH, USA). M-MLV Reverse Transcriptase (Cat#M1705, Promega (Beijing) Biotech Co., Beijing, China) was used to generate cDNA by the RNA template according to the manufacturer's instructions. Quantitative real-time polymerase chain reaction (RT-PCR) was used to determine the mRNA level in the cells. ChamQ SYBR qPCR Master Mix (Cat# Q341-03, Vazyme, Nanjing, China) was used to conduct RT-PCR on a StepOne Plus Real-Time PCR Systems (Cat# 4379216, Thermo Fisher Scientific, California, USA) and analyzed with its own software. The RNA levels of EVs were determined by droplet digital PCR (DD-PCR). The DD-PCR system was developed by Forevergen (Guangzhou, China). The droplets were generated using a 60 *μ*l volume reaction system (40 *μ*l of droplet generation oil) for EvaGreen and 20 *μ*l PCR mixtures, which contained the EvaGreen premix (Cat# S02000201, Forevergen), forward and reverse primers, and the cDNA template, on a droplet generator (MicroDrop-100A, Forevergen). The PCR was conducted on a normal PCR instrument (ETC 811, Eastwin Scientific Equipment Inc., Suzhou, China). Target amplification was analyzed on a droplet reader (MicroDrop -100B, Forevergen) with QuantDrop V1 software. The primers are provided in Table 1.

### 2.8. Enzyme-Linked Immunosorbent Assay

The cytokine levels in the culture medium were detected by ELISA (enzyme-linked immunosorbent assay), and commercialized kits were purchased from Cusabio Technology LLC (Wuhan, Hubei, China), including IL10 (Cat# CSB-E04593 h) and TNF-*α* (Cat# CSB-E04740 h).

### 2.9. Immunofluorescence

The positive rate and intensity of protein expression were detected by immunofluorescence. The primary antibodies were anti-CD11B (Cat# 66519-1-Ig, ProteinTech, Wuhan, China) and anti-CD163 (Cat# 16646-1-AP, ProteinTech), and Alexa Fluor 488 Dnk secondary antibody was used for visualization (Cat# A21206 for anti-Rabbit, Cat# A21202 for anti-Mouse, Invitrogen, Carlsbad, CA, USA). The mean fluorescence intensity (MFI) of in situ immunofluorescence was assayed by the ImageJ program (Version 1.48v, Wayne Rasband, USA).

### 2.10. Western Blot

Western blotting was performed to detect the intracellular proteins. The proteins of different THP-1 treatment groups were harvested using the RIPA (radioimmunoprecipitation assay) lysis buffer, and the concentration was determined by a BCA Protein Assay Kit. Sodium dodecyl sulfate-polyacrylamide gel electrophoresis (SDS-PAGE) was used to distinguish the proteins by their KD size, and the quantity was determined by the gray level of banding. The primary antibodies were anti-phospho-P65 (S536) (Cat# 3033, Cell Signaling Technology, Shanghai, China), anti-total-p65 (Cat# ab7970, Abcam, Cambridge, UK), and anti-iNOS (Cat# 821505270, GeneTex, Beijing, China), and anti-GAPDH was used as a control (Cat# 60004-1-lg, ProteinTech). The secondary antibodies were purchased from Jackson ImmunoResearch Inc. (Cat# 111-035-003 for anti-Rabbit, Cat# 115-035-003 for anti-Mouse, West Grove, PA, USA).

### 2.11. Statistical Analysis

The quantitative results were expressed by histogram according to the means ± SDs. The comparisons of two groups were statistically analyzed using unpaired Student's *t*-tests, and multiple comparisons (over two groups) were performed using ordinary one-way ANOVA tests. The *P* values were determined by follow-up tests, which compared the mean of each column with the mean of every other column. Differences were considered statistically significant at *P* values < 0.05.

## 3. Results

### 3.1. EVs Derived by PEG Had More Particles and Protein than UC

**Figure 1:**
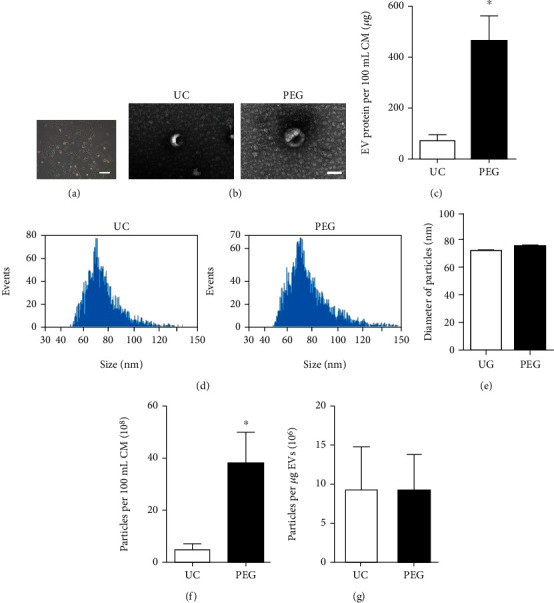
(a) Comparison of the quantity of EVs harvested by UC or PEG. (b) AF-MSCs, which are sources of EV production; *scale* *bar* = 100 *μm*. EVs derived by UC and PEG presented similar morphology by electron microscopy, *scale* *bar* = 100 *nm*. (c) The proteins of EVs were quantified by BCA, and more proteins were derived by PEG than by UC (*n* = 6, ^∗^ for *P* < 0.05). (d) The distribution of EV sizes derived by UC or PEG. (e) There was no difference in the diameter of the particles (*n* = 3), (f) while significantly more particles were harvested by PEG than by UC (*n* = 3, ^∗^ for *P* < 0.05), (g) and there was no difference in particles per *μ*g protein between UC-EVs and PEG-EVs.

The AF-MSCs were subcultured from passage 2 to passage 5, and they could differentiate into mesodermal mature cells and inhibit PHA-activated PBMCs as previously described [19]. The culture medium of AF-MSCs was used to harvest the EVs (Figure 1(a)) by the PEG or UC method. The morphology of the EVs observed by electron microscopy was similar to that of cups or plates derived by UC and PEG (Figure 1(b)). The parallel control experiments were conducted for comparison, as the medium was equally divided into two groups for EVs harvested by UC or PEG. The protein level of each EV harvest was measured by BCA assay, and PEG obtained more proteins than the UC group, *P* = 0.0013 (Figure 1(c)). The size and quantity of EVs were determined by the nanoflow cytometry analysis. The size of EVs ranged from 30 nm to 150 nm, while the distributions of UC and PEG were similar (Figure 1(d)) with no difference in particle diameter (Figure 1(e)). Thus, there were more particles derived from PEG than from UC, *P* = 0.023 (Figure 1(f)), and no difference in particles per *μ*g protein between UC-EVs and PEG-EVs (Figure 1(g)).

### 3.2. Low RNA Levels of EVs Derived by PEG Compared with UC

**Figure 2:**
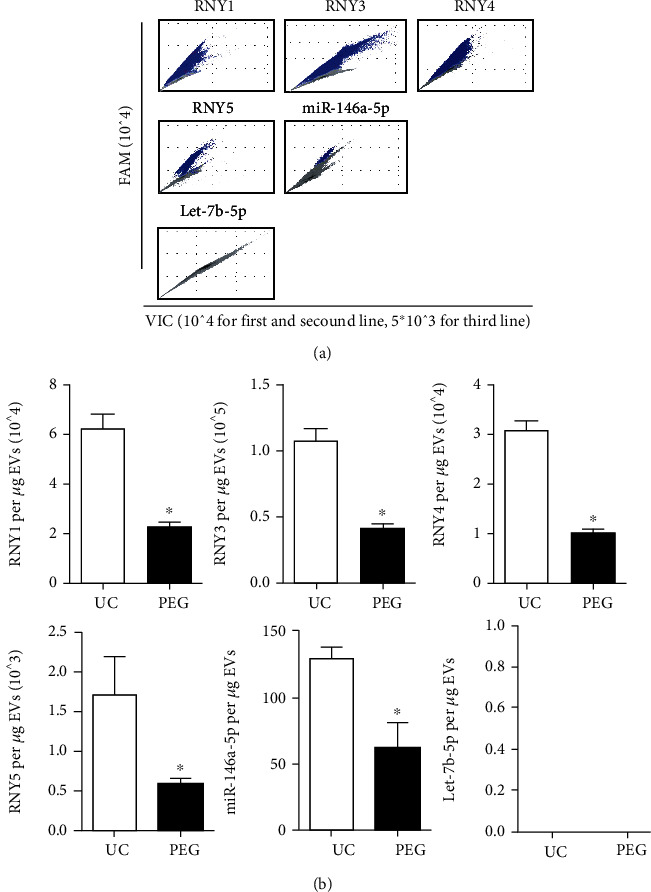
Comparison of RNA expression of UC-EVs and PEG-EVs. (a) The copy numbers of RNY1, RNY3, RNY4, RNY5, miR-146a-5p, and Let-7b-5p detected in EVs by droplet digital PCR; blue points indicate positive reads, and gray points indicate negative reads. (b) the statistical analysis of RNA expression (*n* = 3). The concrete level of each RNA marker was statistically analyzed by the positive copy number reads of droplet digital PCR and its loading quantity. The RNA level of each gene was different by an order of magnitude; the most highly expressed was RNY3, and no Let-7b-5p signal was detected. There was a significant increase in the detected RNA of UC-derived EVs compared with PEG, ^∗^ for *P* < 0.05.

The RNA levels were determined by DD-PCR, and nanoflow cytometry analysis was used to determine the protein levels. The levels of six RNA markers were assayed (Figure 2(a)). The top RNA expression marker was RNY3, followed by RNY1 and RNY4, followed by RNY5 and miR-146a-6p, and Let-7b-5p was not detected. The RNA levels obtained by UC were significantly higher than those obtained by PEG, including RNY1 (*P* = 0.0012), RNY3 (*P* = 0.00067), RNY4 (*P* = 0.000027), RNY5 (*P* = 0.0082), and miR-146a-5p (*P* = 0.0026), while there was no comparison of Let-7b-5p (Figure 2(b)).

### 3.3. No Difference in the Percentage of EV-Specific Markers between UC-EVs and PEG-EVs

**Figure 3:**
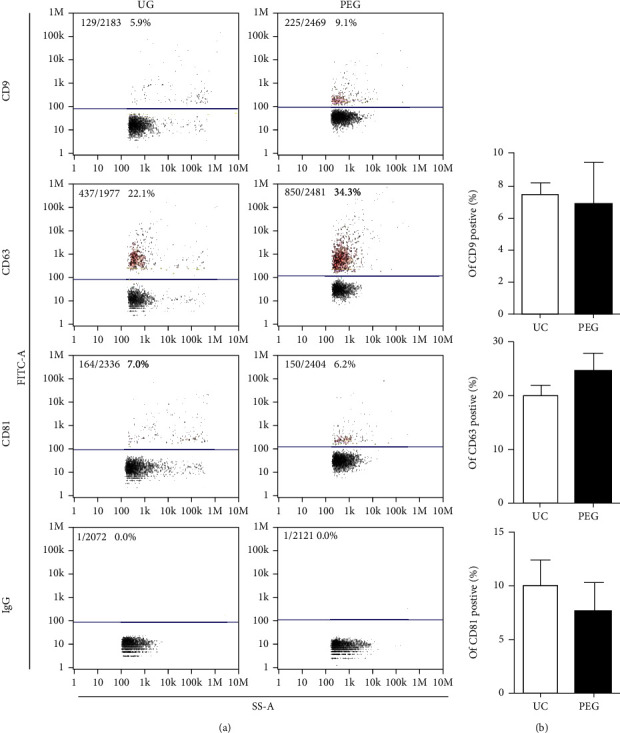
Comparison of the protein expression of UC-EVs and PEG-EVs. (a) The rate of protein expression by nanoflow cytometry analysis, including CD9, CD63, and CD81. IgG was used as a negative control. (b) Statistical analysis indicated that there were no significant differences between the UC and PEG groups (*n* = 4).

Three reported protein markers of EVs were assayed (Figure 3(a)). The top expression marker was CD63, as over twenty percent of the particles were positive, and ten percent of the particles were positive for CD9 and CD81. There were no differences found between UC and PEG (Figure 3(b)). However, PEG harvested more positive particles of selected markers (CD9, CD63, and CD81) in comparison to UC, as the total protein level was higher in the PEG-EVs than in UC-EVs.

### 3.4. EVs Derived from AF-MSCs Inhibit LPS-Induced THP-1 Cytokine Secretion

**Figure 4:**
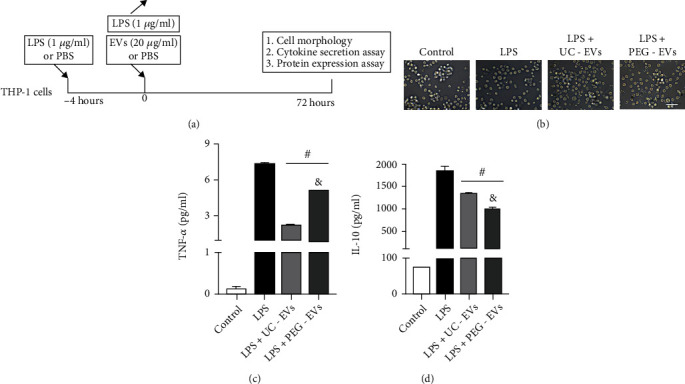
Comparison of EV biological effects on LPS induction of THP-1 cells. (a) Schematic representation of the EV cellular effect assay on the cell model of THP-1 cells treated with LPS. (b) The indicated cell morphology of THP-1 cells treated with LPS and EV; *scale* *bar* = 50 *μm*. Statistical analysis of (c) TNF-*α* and (d) IL-10 secretion indicated a significant reduction by EV treatment compared with the LPS group (^#^ compared with the LPS group and *P* < 0.05) and a difference in the comparison of UC-EVs and PEG-EVs (^&^ compared with the LPS + UC-EVs group and *P* < 0.05).

The biological function of EVs derived from AF-MSCs was verified in a cell model of THP-1 induction by LPS (Figure 4(a)). THP-1 cells were pretreated with 1 *μ*g/ml LPS for 4 h, LPS was then removed, and 20 *μ*g/ml EVs were added to the cell microenvironment by changing the culture medium. Biological analyses were conducted at 72 h, including cell morphology, cytokine secretion, and protein expression assays. For cell morphology, the cell agglomeration phenomenon was partly lost by LPS induction, while EVs altered those cell growth changes (Figure 4(b)). Both UC-EVs and PEG-EVs reduced LPS-induced TNF-*α* and IL-10 secretion, and PEG-EVs induced more TNF-*α* secretion (*P* < 0.0001) and less IL-10 secretion (*P* = 0.0006) than UC-EVs (Figures 4(c) and 4(d)).

**Figure 5:**
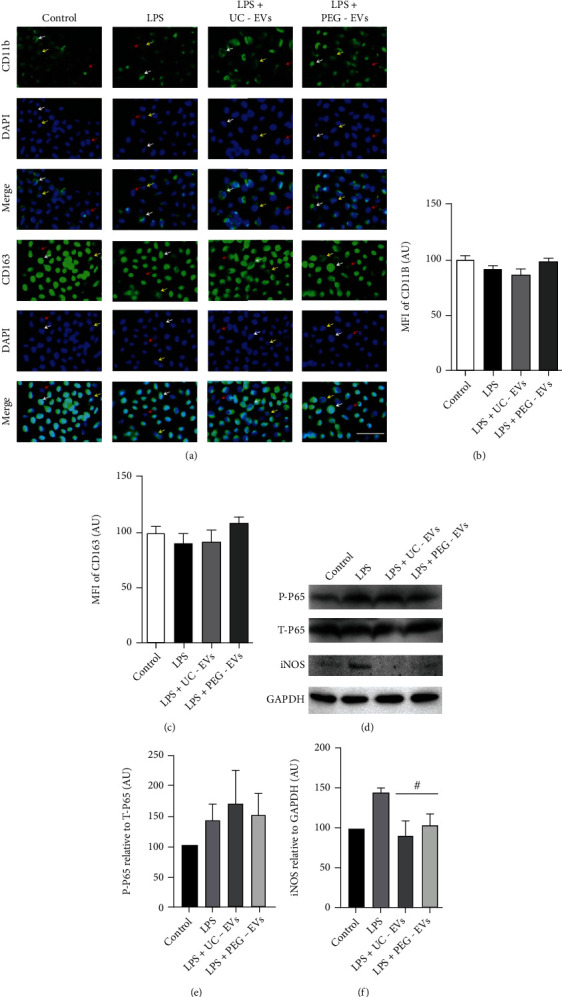
EV treatment leads to changes in the protein expression of THP-1 cells with LPS induction. (a) The in situ CD11b and CD163 expression of THP-1 cells was analyzed by immunofluorescence staining, *scale* *bar* = 50 *μm*. There were strong positive cells (white arrow), medium positive cells (yellow arrow), and negative cells (red arrow) in each group. The statistical analysis of MFI (mean fluorescence intensity) indicated that there were no significant differences in (b) CD11b and (c) CD163 in the groups. (d) Western blotting was used to determine the protein levels of phospho-P65, total-p65, and iNOS, and GAPDH was used as a control. The statistical analysis of protein levels indicated that iNOS relative to GAPDH was significantly decreased by EV treatment (# compared with the LPS group and *P* < 0.05), but there were no significant differences in (e) P-P65 relative to T-P65 and (f) iNOS relative to GAPDH in comparison to UC-EVs and PEG-EVs.

The immunofluorescence staining of CD11b and CD163 indicated that there were strongly positive, medium positive, and negative cells in each group (Figure 5(a)), and significant changes in THP-1 cells were not induced by LPS or EV treatment (Figures 5(b) and 5(c)). LPS induced an increase in phospho-P65 and iNOS expression, but not total p65, compared with the control group; MSCs-EVs induced a decrease in iNOS expression, but not phospho-P65, compared with the LPS group (Figure 5(d)), but there was no significant difference between UC-EV and PEG-EV treatment (Figures 5(e) and 5(f)).

## 4. Discussion

EVs are nanoscale particles that contain nucleic acids and proteins from their cells of origin. EVs are widely accepted as potential biomarkers in medical science for many diseases, including cancer [30]. The other usage of EVs involves replacing their origin cells to generate corresponding biofunctions, such as those EVs derived from MSCs [4, 7]. In the literature, the most commonly used method for EV isolation is UC; the advantage is that it can isolate high-purity EVs from large-volume samples at a low cost [15]. The disadvantage is that expensive instruments (large centrifuge) are essential, and the harvest ratio is low. The other approved method for EV isolation is PEG-based precipitation. The principle of PEG is that EVs are coincubated and then centrifuged at a low speed [31]. The disadvantage is that PEG as a precipitating agent can destroy EV construction derived from plasma [12]. However, another study showed that PEG did not damage EVs derived from saliva [15], and inconsistent results might be due to EVs from different body fluids and PEG concentrations. Furthermore, EVs obtained by UC or PEG also showed differences in morphology and protein levels [15, 31]. In this study, we demonstrated that EVs derived from AF-MSCs by UC or PEG did not differ in size or morphology, while PEG methods produced more EV proteins and particles than UC.

Further comparison studies of EVs derived by UC or PEG were conducted on nucleic acid and protein expression. According to RNA profile data, compared with MSCs, the expression of miRNAs in MSC-EVs was downregulated, and protein-coding RNA levels were upregulated [11]. Y RNAs are small noncoding RNAs and are identified as the RNA component of soluble ribonucleoproteins (RNPs), called Ro RNPs, including human RNY1, RNY3, RNY4, and RNY5 [32]. RO60 is the best-known partner of Y RNAs involved in protein assembly, which is related to the cellular response to environmental stress and is considered a potential prognostic biomarker of immunity [33] and cancer [34]. We examined the expression of Y RNAs in human AF-MSCs-EVs because Y RNAs are potential functional factors of MSCs-EVs and one of the most common families of EVs achieved from equine adipose-derived mesenchymal stromal cells [35]. The other two assayed indices were miR-146a and Let-7b, due to evidence of both being important in MSC-EV biofunction, while EVs derived from the overexpression of miR-146a MSCs effectively ameliorated experimental colitis [36], and Let-7b induced by LPS preconditioning enhanced MSC-EV ability to modify macrophage polarization for the resolution of chronic inflammation [37]. DD-PCR was designed for quantitative analysis of very small samples and has already been used for EV mRNA assays [38]. In this study, we found that Let-7b was not detected in untreated AF-MSCs-EVs, all detected markers showed a similar trend, and the RNA level of UC-EVs increased by 2- to 3-fold compared with PEG-EVs. It is worth noting that in the same MSC medium, PEG resulted in a 6- to 7-fold increase in EV particles and protein harvest compared with UC, which means that in the same MSC medium, PEG resulted in a higher recovery of RNA than UC. The molecular markers of EVs can be analyzed by microfluidics-based detection technologies [39, 40]. CD9, CD63, and CD81 are members of the tetraspanin family [41], and the positive ratio of UC and PEG-EVs was analyzed in this study. However, they are centrifugation-based separation techniques regardless of UC or PEG, and it is difficult to achieve highly purified EVs compared with immunoaffinity capture methods [42]. The positive surface marker ratio of our MSC-EVs was only 10%-20%, and no difference was found between UC-EVs and PEG-EVs.

A cell model of THP-1 cells exposed to LPS [43] was used to evaluate the immunomodulatory effect of EVs. First, the current result of bright field observation of LPS and EV treatment of THP-1 cells indicated that LPS-induced cell spheres are reduced and EVs relieve this phenomenon. The following assay was conducted to determine cytokine secretion and protein expression. The cytokine assay showed accumulation, and the protein assay showed cell expression 72 h after THP-1 exposure to LPS. In the cytokine secretion assay, TNF-*α* is considered a proinflammatory factor, and IL-10 is an anti-inflammatory factor. THP-1 exposure to LPS resulted in an increase in both, and MSC-EVs reversed this phenomenon and even reduced the level of IL10 [44]. EVs of equivalent quality derived by UC or PEG function in different modes of secretion inhibition, and the probable causes could be a result of RNA differences in EVs derived by different methods. CD11B is encoded by the ITGAM gene and is widely expressed in immune cells for functional adhesion, migration, and differentiation [45], and the other protein targets were selected to promote M2 macrophage polarization differentiation according to MSC-EVs functional in inflammatory responses [24], including CD163, phospho-P65, total-p65, and iNOS [21]. Immunofluorescence was used for the relative quantitative analysis of protein expression. Our results showed that there was no significant change in the positive rates of CD163 and CD11b in THP-1 cells in each group, which may be due to insufficient intensity of LPS treatment to induce a change in THP-1 protein expression. Western blotting results showed that there were no differences in protein expression between UC-EV- and PEG-EV-treated THP-1 cells.

## 5. Conclusion

In summary, we derived EVs from the AF-MSCs culture medium using the UC and PEG methods and compared the precipitation methods and EVs. First, UC-EVs and PEG-EVs shared a similar morphology, size, and specific marker-positive ratio. Furthermore, PEG precipitation harvested more EV particles, protein, and RNA than the UC method. Finally, MSC-EVs inhibited LPS-induced inflammatory-associated cytokine increases, and there were differences between UC-EVs and PEG-EVs. Further studies are needed to define the RNA and protein expression profiles of UC-EVs and PEG-EVs and test their biological function in more specific models in vivo and in vitro.

## Figures and Tables

**Table 1:** DD-PCR primers.

RNY1-F	TGTTCACAGTCAGTTACAGATCG
RNY1-R	AGTCAAGTGCAGTAGTGAGAAGG
RNY3-F	TCCGAGTGCAGTGGTGTTTAC
RNY3-R	AGGCTAGTCAAGTGAAGCAGTG
RNY4-F	TCCGATGGTAGTGGGTTATCAG
RNY4-R	AGCCAGTCAAATTTAGCAGTGG
RNY5-F	AGTTGGTCCGAGTGTTGTGG
RNY5-R	TAGTCAAGCGCGGTTGTGG
Let-7b-5p-F	AACACGCTGAGGTAGTAGGTT
Let-7b-5p-R	GTCGTATCCAGTGCAGGGTCCGAGGTATTCGCACTGGATACGACAACCAC
miR-146a-5p-F	TGAGAACTGAATTCCATGG
miR-146a-5p-R	GTCGTATCCAGTGCAGGGTCCGAGGTATTCGCACTGGATACGACAACCCA

## Data Availability

No data were used to support this study.
